# The global landscape of myopia prevalence and its social determinants in children and adolescents: a cross-regional analysis

**DOI:** 10.3389/fopht.2026.1793615

**Published:** 2026-07-13

**Authors:** Chengfei Liu, Wei Dong, Zihao Wang, Yanying Li, Boyang Song, Yukang Li, Ye Zhang, Xiaokang He, Zhaojiang Du

**Affiliations:** 1 Department of Ophthalmology, Xi’an Central Hospital, Xi’an, China; 2 Department of Clinical Medicine, Yan’an University, Yan’an, Shaanxi, China; 3 Department of Ophthalmology, The Second Affiliated Hospital of Xi’an Jiaotong University, Xi’an, China; 4 Department of Ophthalmology, Baoji People’s Hospital, Baoji, China

**Keywords:** demographic structure, economic development, education, geographical environment, myopia prevalence, social welfare

## Abstract

**Background:**

Investigation of the global profile of myopia prevalence in children and adolescents and analysis of the impact of social factors, including economic development level, social welfare, education, demographic structure, and geographical environment, on the progression of myopia.

**Methods:**

A retrospective study analyzed data from 31 global datasets (2004-2023) to assess social determinants of myopia prevalence. Using univariate and multiple linear regression, we evaluated the associations between myopia prevalence in children and adolescents and macroeconomic, educational, demographic, and environmental factors sourced from public databases. To further assess the robustness of the results, sensitivity analyses were conducted.

**Results:**

Univariate linear regression analysis revealed that GDP (β = 0.369, *P* = 0.027, R^2^ = 0.128) and longitude (β = 0.551, *P* = 0.001, R^2^ = 0.280) are significantly positively correlated with myopia prevalence. The multiple linear regression model demonstrated a good overall fit (R = 0.834, R^2^ = 0.695, adjusted R^2^ = 0.565), and in this model, GDP per capita was negatively associated with myopia prevalence (β = –0.863, *P* = 0.001), whereas population distribution in the Northern and Southern Hemispheres (β = 0.572, P = 0.011), urban population (% of total population) (β = 0.737, *P* = 0.008), and longitude (β = 0.508, *P* = 0.006) were significantly positively correlated with myopia prevalence (*P* < 0.05).The negative associations of GDP per capita and average sunshine duration with myopia prevalence remained directionally consistent across most sensitivity analyses.

**Conclusion:**

East Asia exhibits the highest global prevalence of myopia in children and adolescents, while Europe and the Americas demonstrate comparatively lower, though variable, rates. Key influencing factors include economic development (GDP), social welfare (GDP per capita), demographic structure (population distribution and urbanization), and geographical setting (longitude).

## 1 Introduction

Myopia prevention and control have emerged as critical issues in the field of global public health in recent years. The prevalence of myopia among children and adolescents continues to rise worldwide, with recent projections indicating that the global myopia prevalence will reach 45.20% by 2040 and 49.80% by 2050 (1). There is clear epidemiological evidence indicating that the prevalence of myopia is increasing most rapidly in Southeast Asia, with projections suggesting that the rate in Asia will reach 68.78% by 2050 (2). Evidence suggests that the onset of myopia is influenced by the interplay of multiple factors. In addition to the non-modifiable factors such as age, ethnicity, and genetics, certain external variables such as environmental factors, socioeconomic status, and public policy also play significant roles (3). Therefore, in this study, the literature was systematically reviewed to summarize the current status of myopia prevalence across different global regions. By integrating the macro-level data on economic development, social welfare, education, the demographic structure, and the geographical environment available in public databases, this study aimed to thoroughly analyze the societal factors influencing myopia prevalence, thereby providing a scientific basis for developing targeted prevention and control strategies for this condition.

## 2 Materials and methods

### 2.1 Study cohort and regional scope

This study is based on the myopia prevalence data (2004–2023) obtained from 20 distinct regions spanning 19 countries worldwide. Definition of myopia: a condition in which the spherical equivalent refractive error of an eye is ≤ -0.50 D when ocular accommodation is relaxed. The cohort used in this study comprised the following geographical distribution: 8 regions in 7 countries from Asia, namely, mainland China, Hong Kong (China), Japan, Korea, India, and Singapore; 7 countries from Europe: Russia, Denmark, the United Kingdom, Germany, France, Spain, and Italy; 5 nations from the Americas: Canada, the United States, Mexico, Argentina, and Brazil. Data from Africa and Australia were also included. Using the available data on myopia prevalence across different years, a final collection of 31 sample datasets was included in this study and used for subsequent statistical analysis.

### 2.2 Data collection and sources

In order to analyze the impact of social factors on myopia prevalence, a retrospective study was employed. This study defined five core dimensions and their corresponding operationalized indicators: 1) economic development level, measured by gross domestic product (GDP); 2) social welfare, represented by GDP per capita; 3) education, characterized by higher education enrollment rate and government expenditure on education, total (% of government expenditure); 4) demographic structure, population distribution in the Northern and Southern Hemispheres, urban population (% of total population), and female population (% of total population); 5) geographical environment, indicated by longitude and average daily sunlight duration.

All the above data were matched and extracted by country and year from the publicly accessible online databases. The myopia prevalence data were systematically retrieved from the PubMed database and other relevant databases using search strings that combined specific region names with the keyword “myopia prevalence”. Data for all the social factors to be evaluated in this study, including economic, educational, demographic, and environmental metrics, were rigorously sourced from the internationally recognized and authoritative public databases, primarily the World Bank.

### 2.3 Statistical analysis

Data analysis was performed using SPSS 27.0. Univariate and multiple linear regression analyses were performed to determine the associations between various social factors and the prevalence of myopia. Among the independent variables, a binary categorical variable—population distribution in the Northern and Southern Hemispheres—was included. This variable was entered into the regression model as a dummy variable, coded as 0 for the Southern Hemisphere (reference category) and 1 for the Northern Hemisphere (exposed category). All other independent variables were continuous and entered into the regression analysis using their original measured values. And the regression model was diagnosed using the normal P-P plot of residuals and the scatter plot of residuals versus predicted values. The results showed that the residuals approximately followed a normal distribution, satisfying the prerequisite assumptions for linear regression analysis. The goodness-of-fit of each regression model was evaluated based on the coefficient of determination (R^2^), and its statistical significance was assessed using the F test. The significance of the individual regression coefficients was tested using the t-test, with a significance level set at α = 0.05. In order to ensure model stability, multicollinearity among the independent variables was evaluated by calculating tolerance and the variance inflation factor (VIF).The diagnostic criteria were as follows: tolerance > 0.1 and VIF < 10 indicated the absence of serious multicollinearity. A two-tailed P < 0.05 was considered statistically significant. To further test the robustness of the model, sensitivity analyses were conducted using Stata. This involved altering the form of the GDP variable, excluding extreme myopia prevalence data, additionally adjusting for education expenditure, female population proportion, and Northern/Southern Hemisphere factors, restricting the analysis to data from 2010 onwards, and employing robust regression.

## 3 Results

### 3.1 Global distribution of myopia prevalence

Asia was identified as a high-prevalence region for myopia, with people in East Asia being particularly affected by this condition. The reported rates were notably high: in mainland China (2020–2022), the prevalence rates among children and adolescents were 52.70%, 52.60, and 51.90%, respectively (4, 5). In Hong Kong, China (2021), 36.20% of children aged 6–8 years were myopic (6). Similarly, in Japan (2017), 76.50% of elementary high school students and 94.90% of junior high school students were affected (7). The prevalence was 58.90% among 19-year-old male military conscripts in Korea (2020) (8), whereas prevalence rates in India (2022) and Singapore (2018) for the children and adolescents aged 5–16 and 8–9 years were 34.79% (9) and 37.30%, respectively (10).

The European population presented rates that were generally lower but with considerable variation. A high prevalence of 65.60% was reported in Russian adolescents aged 17–18.8 years (2019–2022) (11), and a high prevalence of 40.56% was reported in Italian children aged 5–12 years (2021) (12). Moderate rates were observed in the Denmark population (25.00% in 16–17-year-old subjects, 2016–2017) (13) and Spain (16.80–20.40% in 5–7-year-old subjects, 2016–2019) (14). A lower prevalence was recorded in the United Kingdom (16.40% in 12–13-year-old subjects, 2016) (15), Germany (11.40% in individuals aged 0–17 years, 2014–2017) (16), and France (15.50% in 2–12-year-olds, 2015–2018) (17).

In Africa, the prevalence of myopia among school-aged children (2021–2023) ranged from 4.70% to 16.05%, with higher rates typically observed in the 10–18 year age group (18, 19).

Data from the Americas revealed a prevalence of 41.60% in the United States (1999–2004) for individuals aged 12–54, representing an almost doubled rate over the preceding three decades (20). In Mexico (2013–2014), 31.20% of the children aged 6–12 years were myopic (21), and the prevalence among 16–17-year-old adolescents in Argentina was 34.10% (2023) (22). Lower rates were reported in Canada (17.50% in children aged 6–8 and 11–13 years, 2018) (23) and Brazil (17.40% in individuals aged 5–20 years, 2019–2021) (24).

**Figure 1:**
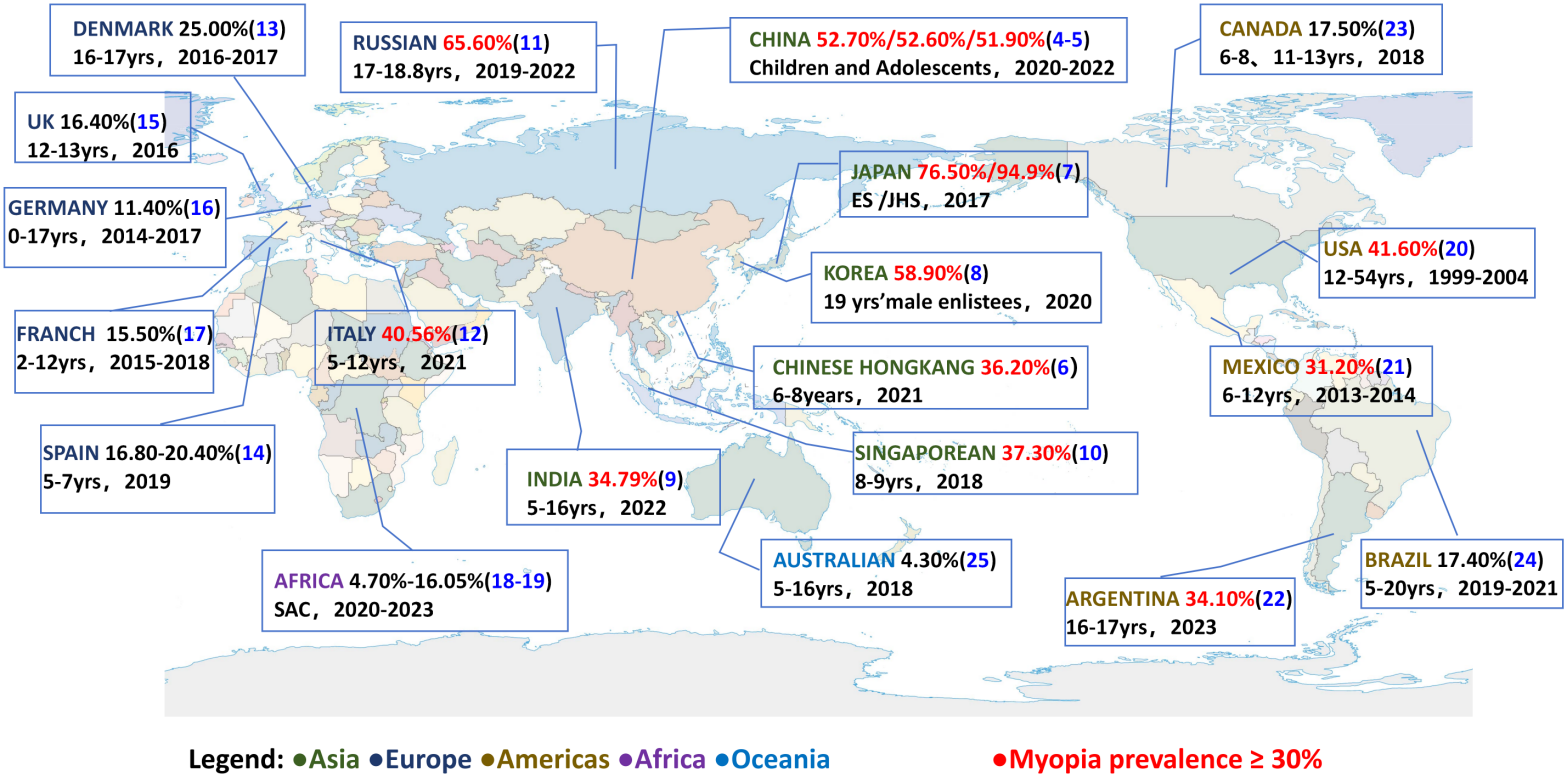
Summary of the prevalence of myopia in different regions of the world. Data points represent reported myopia prevalence (%) with corresponding age groups and survey years. Colors indicate continent classification: Asia (green), Europe (dark blue), Americas (brown), Africa (purple), and Australia (light blue). Data points with myopia prevalence ≥ 30% are shown in red.

Oceania, which was represented by Australia in this study, reported the lowest prevalence, at just 4.30% among children aged 5–16 years (2018) (25) (**Figure 1**; **Table 1**).

**Table 1:** Characteristics of included studies on myopia prevalence across different regions.

Region	Year	Age range	Survey location	Sample size	Cycloplegia	Refraction assessment method
Mainland China	2020-2022	Children and adolescents	School	Nationwide survey	No	Automated refraction
Hong Kong (China)	2021	6–8 years	Hospital	20,527	Yes	Automated refraction
Japan	2017	Elementary and junior high school students	School	1,478	No	Automated refraction
Korea	2020	19-year-old male military conscripts	Hospital	2,215,126	No	Automated refraction
India	2022	5–16 years	School	1,400	No	Retinoscopy
Singapore	2018	8–9 years	Hospital	572	Yes	Automated refraction
Russia	2019-2022	17-18.8 years	School	4,933	Yes	Automated and Subjective refraction
Italy	2021	5–12 years	Hospital	803	Yes	Retinoscopy
Denmark	2016-2017	16–17 years	–	1,443	No	Automated and Subjective refraction
Spain	2016-2019	5–7 years	School	9,668	No	Automated and Subjective refraction
United Kingdom	2016	12–13 years	School	669	Yes	Automated refraction
Germany	2014-2017	0–17 years	–	15,023	–	–
France	2015-2018	2–12 years	Hospital	48,163	Yes	Automated/Subjective refraction
Africa	2020-2023	School-aged children	–	10,031-36,395	–	–
United States	1999-2004	12–54 years	–	9,609	No	Automated refraction
Mexico	2013-2014	6–12 years	School	3,861,156	No	Retinoscopy
Argentina	2013	16–17 years	Hospital	384	Yes	Automated refraction
Canada	2018	6-8, 11–13 years	School	166	Yes	Automated and Subjective refraction
Brazil	2019-2021	5–20 years	School	330	No	Automated and Subjective refraction
Australia	2018	16–17 years	School	4,365	No	Retinoscopy

Data for Africa were compiled from different literature sources, with sample size presented as a range. Dashes (–) indicate information not available.

### 3.2 Univariate linear regression analysis of the factors influencing myopia prevalence

**Figure 2:**
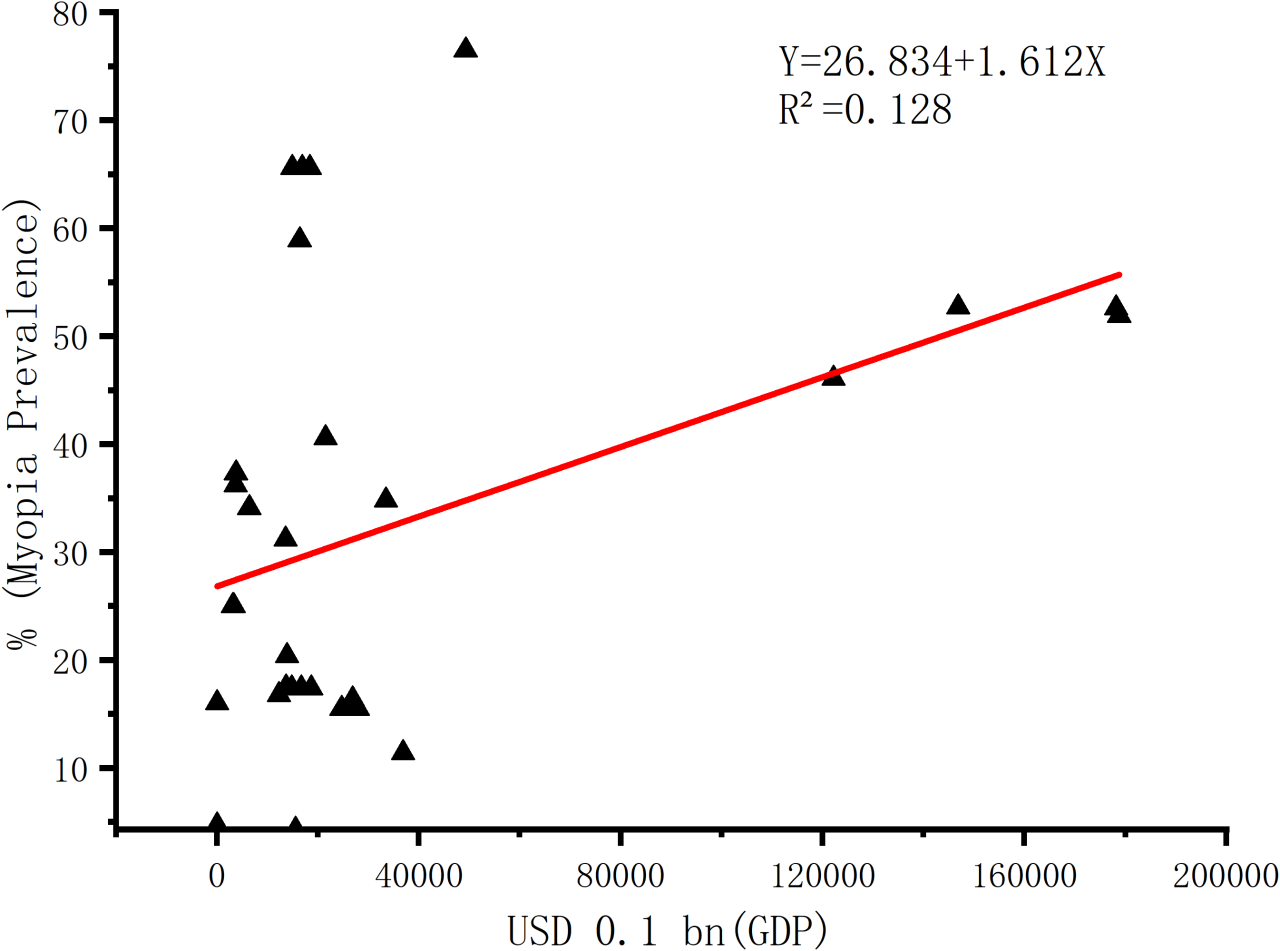
Myopia significantly increased with GDP. The data show a significant positive linear correlation (black scatters). The red solid line is the fitted linear regression line (equation: Y = 26.834 + 0.612X, R² = 0.128, p < 0.05; n=31).

**Figure 3:**
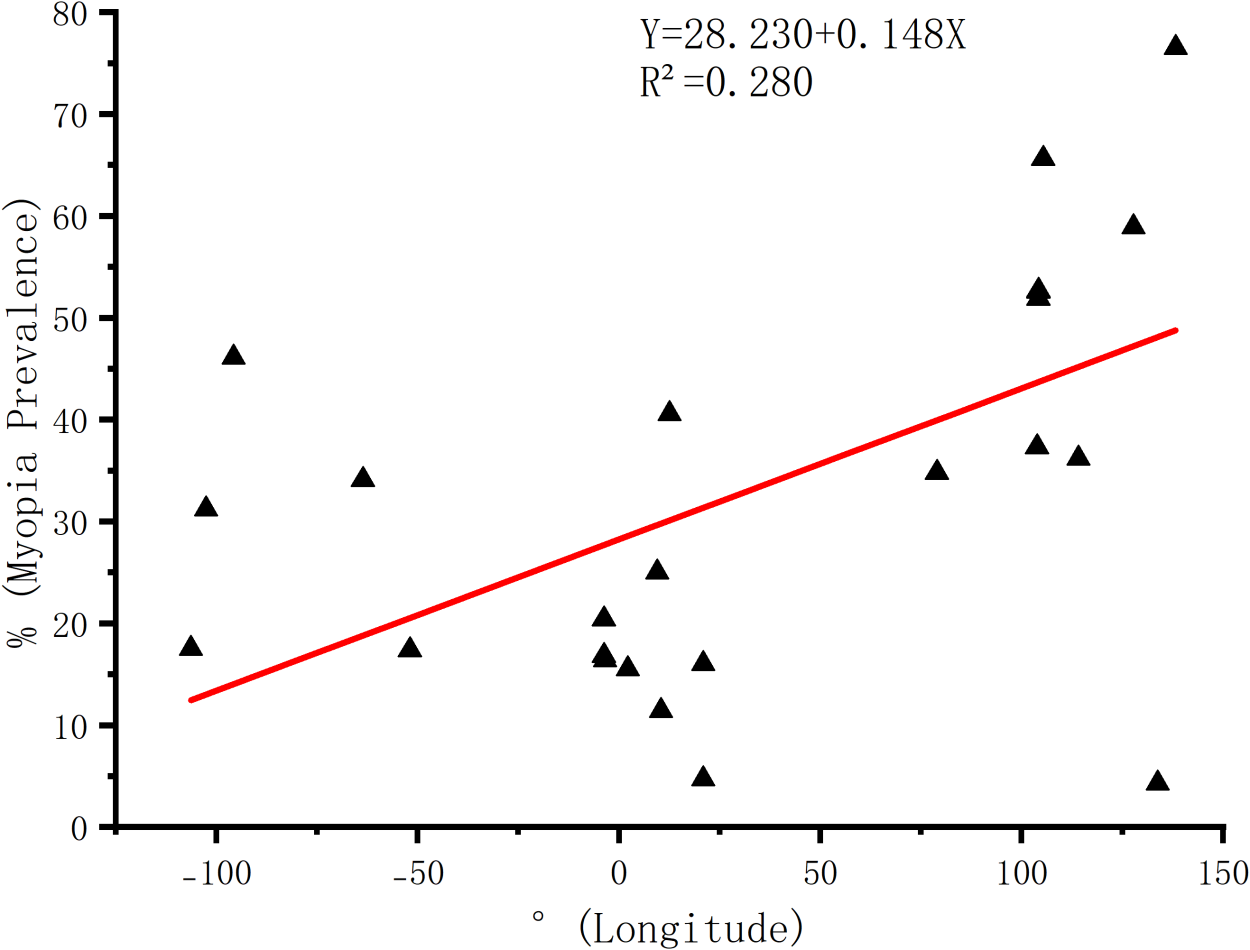
Myopia significantly increased with longitude. The data show a significant positive linear correlation (black scatters). The red solid line is the fitted linear regression line (equation: Y = 28.230 + 0.148X, R² =0.280, p < 0.05; n=31).

The univariate linear regression analysis identified two factors with statistically significant associations with myopia prevalence. Both GDP (β = 0.369, *P* = 0.027, R^2^ = 0.128) and longitude (β = 0.551, *P* = 0.001, R^2^ = 0.280) demonstrated significant positive correlations (**Figures 2**, **3**, respectively).

In contrast, the regression models for the remaining variables did not reach statistical significance (*P* ≥ 0.05). A negative, non-significant association with myopia prevalence was observed for GDP per capita (β = –0.177, *P* = 0.342), average sunlight duration (β = –0.351, *P* = 0.053), and government expenditure on education in total (% government expenditure) (β = –0.264, *P* = 0.151). Similarly, no significant positive associations were found with a higher education enrollment rate (β = 0.157, *P* = 0.399), population distribution in the Northern and Southern Hemispheres (β = 0.316, *P* = 0.083), the urban population (% of the total population) (β = 0.008, *P* = 0.966), and the female population (% of the total population) (β = 0.122, *P* = 0.512).

### 3.3 Multivariate linear regression analysis of the factors influencing myopia prevalence

The multivariate linear regression model was statistically significant (F = 5.325, *p* < 0.001) and demonstrated a good fit, with an R of 0.834, R^2^ of 0.695, and an adjusted R^2^ of 0.565. Among the social factors analyzed, several were identified as independent predictors of myopia prevalence with significance. The GDP per capita exhibited a significant negative association (β = –0.863, *p* = 0.001). In contrast, the population distributions in the Northern and Southern Hemispheres (β = 0.572, *p* = 0.011), the urban population (% of the total population) (β = 0.737, *p* = 0.008), and longitude (β = 0.508, *p* = 0.006) were significantly positively associated with myopia prevalence (*p* < 0.05).

The associations of the remaining variables with myopia prevalence, including GDP (β = 0.186), the gross enrollment ratio in tertiary education (β = 0.104), government expenditure on education in total (% government expenditure) (β = 0.076), the female population (% of total population) (β = –0.083), and average sunlight duration (β = –0.047) did not reach statistical significance (*p* ≥ 0.05).

**Figure 4:**
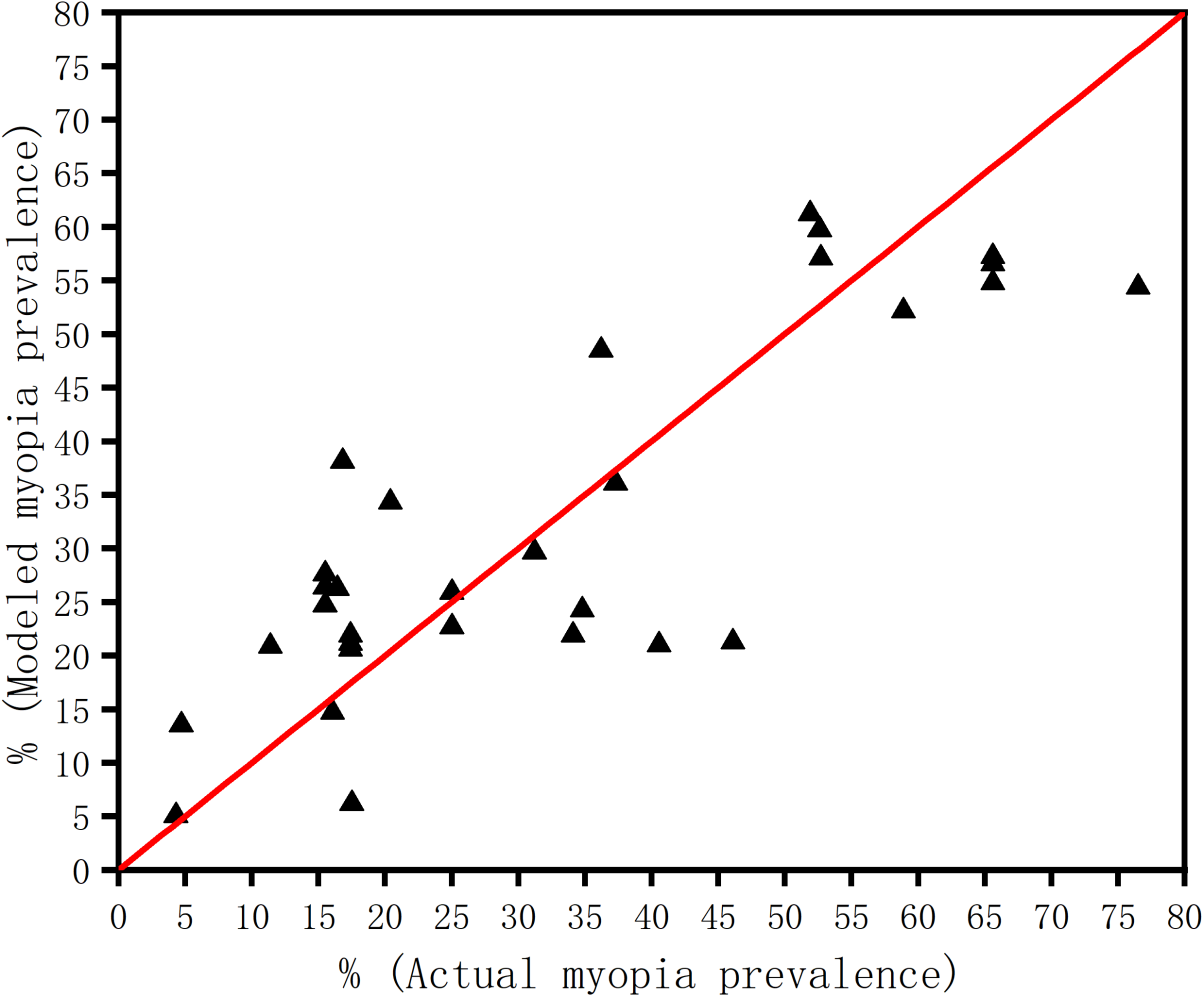
Observed versus predicted values of Myopia Prevalence from the multiple linear regression model. The solid red line represents the line of perfect agreement (y = x). The black scatters shows the linear fit through the data points (R² =0.565, p <0.001; n =31). The model included the following predictors: GDP, GDP per capita, Higher Education Enrollment Rate, Government expenditure on education, total (% of government expenditure), population distribution in the Northern and Southern Hemispheres, Urban population (% of total population), Female population (% of total population), Longitude, and Average Sunlight Duration.

Detailed results of the multivariate linear regression analysis are summarized in **Table 2** and further illustrated in **Figure 4**.

**Table 2:** Multiple linear regression analysis of the factors associated with myopia prevalence.

	Unstandardized coefficient	Standardized coefficient					
Predictor	B	Standard error	β	t	p	Tolerance	VIF
(Constant)	-1.145	134.770		-0.008	0.993		
GDP	7.551	0.000	0.186	1.131	0.271	0.539	
GDP per capita	-0.001	0.000	-0.863	-3.772	0.001	0.277	1.855
Higher Education Enrollment Rate	0.084	0.167	0.104	0.503	0.620	0.340	3.611
Government expenditure on education, total (% of government expenditure)	1.230	2.779	0.076	0.443	0.663	0.489	2.937
Population distribution in the Northern and Southern Hemispheres	30.885	11.056	0.572	2.794	0.011	0.346	2.045
Urban population (% of total population)	0.981	0.337	0.737	2.908	0.008	0.226	2.889
Female population (% of total population)	-1.113	2.399	-0.083	-0.464	0.647	0.453	4.432
Longitude	0.137	0.045	0.508	3.034	0.006	0.518	2.210
Average Sunlight Duration	-0.634	2.738	-0.047	-0.232	0.819	0.358	1.932

Model with myopia prevalence as the dependent variable (R^2^ = 0.695, adjusted R^2^ = 0.565, F = 5.325, *p* < 0.001).

### 3.4 Sensitivity analysis results for myopia prevalence

Sensitivity analysis results indicated that GDP per capita exhibited a significant negative association in the original models (β ranging from –0.0006 to –0.0009, p < 0.01); however, this association did not reach statistical significance after logarithmic transformation (β = –4.75, p > 0.05). Average sunshine duration was significantly negatively associated in five of the six sensitivity models (β ranging from –10.62 to –4.25), although its significance attenuated in the fully adjusted model (p > 0.05). The significance and direction of the higher education enrollment rate and urban population proportion were inconsistent across the different sensitivity models. Detailed results of the sensitivity analyses are summarized in **Table 3**.

**Table 3:** Sensitivity analysis results for myopia prevalence.

Variable	Main	Log GDP	No extremes	Full model	Year ≥2010	Robust regression
GDP per capita	-0.000618** (0.000173)	—	-0.000657*** (0.000157)	-0.000924*** (0.000212)	-0.000661*** (0.000169)	-0.000882*** (0.000107)
ln(GDP per capita)	—	-4.754 (5.200)	—	—	—	—
Higher Education Enrollment Rate	0.295 (0.214)	0.274 (0.281)	0.412* (0.188)	0.311 (0.201)	0.274 (0.214)	0.521*** (0.0876)
Government expenditure on education, total (% of government expenditure)	—	—	—	-3.197 (2.789)	—	—
Population distribution in the Northern and Southern Hemispheres	—	—	—	27.76 (14.46)	—	—
Urban population (% of total population)	-0.0697 (0.367)	-0.198 (0.303)	-0.440* (0.208)	0.619 (0.494)	-0.0343 (0.372)	-0.570*** (0.146)
Female population (% of total population)	—	—	—	-2.672* (1.252)	—	—
Average sunshine duration	-6.894** (2.094)	-5.802 (3.042)	-7.795*** (1.975)	-4.252 (3.494)	-7.282** (2.064)	-10.62*** (1.138)
N	31	31	28	31	30	31
R2	0.340	0.180	0.566	0.522	0.369	0.861

Coefficients are shown with standard errors in parentheses. — indicates that the variable was not included in that model. Significance: * p<0.05; ** p<0.01; *** p<0.001.

## 4 Discussion

This study elucidated the complex associations of macroeconomic, demographic, and geographic factors with myopia prevalence in children and adolescents. The multivariate regression model identified several significant independent predictors, thereby offering insights into the multifaceted etiology of myopia.

First, this study revealed a nuanced relationship between the economic indicators and myopia. While higher national GDP was associated with increased myopia prevalence, as revealed in the univariate analysis, the multivariate analysis identified GDP per capita as a factor with a significant protective effect. The positive association with the overall GDP may be linked to the intensified near-work demands within the evolving educational and professional systems, which is a well-established risk factor for myopia (26). Conversely, the higher GDP per capita likely signifies enhanced healthcare infrastructure, greater public health resources, and increased capacity for systemic myopia prevention initiatives, which may include school-based vision screening programs, optimized classroom lighting, public health campaigns promoting outdoor activities, and academic load reduction (27–29). Further, the households with greater disposable income are better positioned to adopt costly interventions such as orthokeratology or specialized lenses to decelerate myopia progression (30). Although the effect sizes were attenuated in the log GDP model and the fully adjusted model during sensitivity analyses—suggesting that the strength of the associations may be influenced by variable scaling and residual confounding—the negative association between GDP per capita and myopia prevalence remained directionally consistent across most sensitivity models, indicating that the primary results are reasonably robust. This divergence underscores the complex interplay of economic development patterns, public policy priorities, and social values in the myopia epidemic. Notably, the higher education enrollment rate exhibited marked instability across the different sensitivity models. This finding also suggests that education remains an important factor that cannot be overlooked in the context of myopia prevalence.

Second, the significant positive associations with longitudinal and Northern Hemisphere population distributions highlight the important geographic and potential socioecological influences. Populations within similar longitudinal ranges share synchronized circadian rhythms and photoperiods, and evidence suggests that circadian disruption can impact the levels of retinal dopamine, a key neurotransmitter that regulates ocular growth, thereby influencing the increase in myopia prevalence (31–33).Of course, the regional distribution of educational pressure cannot be ruled out as a potential confounding factor that interferes with the effect of longitude on myopia prevalence. Further, the densely populated and rapidly urbanizing regions of the Northern Hemisphere often correspond to the lifestyles characterized by insufficient outdoor time and limited access to green spaces for children, which reduces exposure to beneficial high-intensity light that stimulates retinal dopamine release and suppresses axial elongation (34–37). Concurrently, these urban environmental increase exposure to high levels of air pollution, which is implicated in oxidative stress and visual impairment (38, 39).

Third, the significant association between a greater proportion of the urban population and myopia prevalence underscores the role of the constructed environment. Light intensity, spectral composition, and spatial frequency of visual stimuli are markedly different in urban and indoor environments than those in natural outdoor settings (40–42). Although the specific metric of “average sunlight duration” used in this study was not statistically significant in the multivariate model, it showed significant associations in multiple sensitivity analysis models. This finding is consistent with previous studies indicating that light intensity is a protective factor against myopia (36); however, the potential interaction between outdoor activity duration and light intensity should also be considered. Future research should prioritize more precise measurements of individual light exposure and detailed characterization of the visual environment to elucidate the underlying photobiological mechanisms.

## 5 Limitations and future research directions

First, the sources of samples in this study were heterogeneous, and due to objective constraints, age and population composition could not be adequately controlled. This resulted in a wide age range across included individuals, potentially introducing selection bias. Estimates of myopia prevalence were derived from highly diverse sources, including hospital-based cohorts, school-based screenings, nationally representative surveys, and military conscript populations. Second, some of the included studies employed non-cycloplegic refraction methods, a factor that may systematically overestimate myopia prevalence in pediatric and adolescent populations. Third, this study applied national-level macro indicators as explanatory variables for individual-level disease prevalence, an analytical approach that may give rise to ecological fallacies. Fourth, data for the African region were aggregated estimates based on published literature, which may compromise cross-regional comparability. Fifth, the analysis did not incorporate several key confounding factors, such as screen time, near-work duration, outdoor exposure, genetic predisposition, and educational pressure. Furthermore, heterogeneity across the underlying datasets raises the possibility that the conclusions may be at risk of overgeneralization. Constrained by the large geographic span across regions and the inherent difficulties in data integration, we were unable to assemble complete individual-level datasets; consequently, the number of social factors that could be examined, as well as the overall sample size, remained relatively limited.

Despite the above limitations, the findings of this study still reveal overall trends in the association between myopia prevalence and environmental factors, thereby providing some reference value. Future research should include larger and more diverse samples, incorporate a broader set of factors known to influence myopia prevalence, and standardize as well as harmonize the methods used for assessing myopia prevalence, in order to reduce data heterogeneity and mitigate the impact of confounding influences.

## 6 Conclusion

This study reveals a marked geographic disparity in myopia prevalence among children and adolescents, with the highest burden observed in Asia, particularly East Asia, and comparatively lower, yet variable rates observed across Europe and the Americas. The influence of economic factors on myopia is dual-faceted: while an increase in gross domestic product (GDP) is associated with increased myopia prevalence, potentially due to the intensifying near-work demands and accelerating urbanization, an increase in GDP per capita has a protective effect, which is likely mediated by an increased investment in public health and higher accessibility of ocular health resources. Further, the specific demographic structures, namely, the population distribution across hemispheres and the proportion of the urban population, and the geographic factors, such as longitude, were significantly associated with myopia prevalence. Shared biological rhythms and light exposure patterns within specific longitudinal zones may collectively influence myopia development through complex mechanisms. It is possible that variations in the data may have introduced limitations in delineating the role of innate factors, such as race and genetics, in the rising prevalence of myopia; nonetheless, this research provides valuable evidence for identifying the key social and environmental determinants of the myopia epidemic and has significant implications for the formulation of targeted prevention and control strategies for this condition.

## Data Availability

The original contributions presented in the study are included in the article. Further inquiries can be directed to the corresponding “author”.
